# Evaluation of Lentiviral-Mediated Expression of Sodium Iodide Symporter in Anaplastic Thyroid Cancer and the Efficacy of In Vivo Imaging and Therapy

**DOI:** 10.1155/2011/178967

**Published:** 2011-12-15

**Authors:** Chien-Chih Ke, Ya-Ju Hsieh, Luen Hwu, Fu-Hui Wang, Fu-Du Chen, Lee-Shing Chu, Oscar K. Lee, Chin-Wen Chi, Chen-Hsen Lee, Ren-Shyan Liu

**Affiliations:** ^1^Institute of Clinical Medicine, National Yang-Ming University, Taipei 11221, Taiwan; ^2^Molecular and Genetic Imaging Core, NRPGM, Taipei 11221, Taiwan; ^3^Department of Medical Imaging and Radiological Sciences, Kaohsiung Medical University, Kaohsiung 80708, Taiwan; ^4^Faculty of Medicine, National Yang-Ming University, Taipei 11221, Taiwan; ^5^Department of Biotechnology, TransWorld University, Yunlin 64063, Taiwan; ^6^National PET/Cyclotron Center, Taipei Veterans General Hospital, Taipei 11221, Taiwan; ^7^Department of Orthopaedics & Traumatology, Taipei Veterans General Hospital, Taipei 11221, Taiwan; ^8^Stem Cell Research Center, National Yang-Ming University, Taipei 11221, Taiwan; ^9^Institute of Pharmacology, National Yang-Ming University, Taipei 11221, Taiwan; ^10^Division of General Surgery, Department of Surgery, Taipei Veterans General Hospital, Taipei 11221, Taiwan; ^11^Department of Biomedical Imaging and Radiological Sciences, National Yang-Ming University, Taipei 11221, Taiwan; ^12^Department of Nuclear Medicine, National Yang-Ming University Medical School, Taipei 11221, Taiwan

## Abstract

Anaplastic thyroid carcinoma (ATC) is one of the most deadly cancers. With intensive multimodalities of treatment, the survival remains low. ATC is not sensitive to ^131^I therapy due to loss of sodium iodide symporter (NIS) gene expression. We have previously generated a stable human NIS-expressing ATC cell line, ARO, and the ability of iodide accumulation was restored. To make NIS-mediated gene therapy more applicable, this study aimed to establish a lentiviral system for transferring hNIS gene to cells and to evaluate the efficacy of in vitro and in vivo radioiodide accumulation for imaging and therapy. Lentivirus containing hNIS cDNA were produced to transduce ARO cells which do not concentrate iodide. Gene expression, cell function, radioiodide imaging and treatment were evaluated in vitro and in vivo. Results showed that the transduced cells were restored to express hNIS and accumulated higher amount of radioiodide than parental cells. Therapeutic dose of ^131^I effectively inhibited the tumor growth derived from transduced cells as compared to saline-treated mice. Our results suggest that the lentiviral system efficiently transferred and expressed hNIS gene in ATC cells. The transduced cells showed a promising result of tumor imaging and therapy.

## 1. Introduction

Anaplastic thyroid cancer (ATC) is rare and represents less than 2% of all thyroid cancer [1]. Nevertheless, it is one of the most deadly human cancers which causes more than half of the deaths attributed to thyroid cancer [2]. Currently, treatment for ATC is mainly surgery combined with chemotherapy and radiotherapy. Although intensive multimodalities of treatment shows improved local control, the overall survival remains low [3]. Other strategy for treating ATC including new drugs is urgently underway.

ATC does not concentrate iodide due to the loss of expression of sodium iodide symporter (NIS) gene. NIS is a membrane protein with 13 putative transmembrane domain [4]. This symporter is driven by low internal sodium concentration and symports 2 sodium ions for every iodide ion into cells. For thyroid cells, NIS mediated iodide transport is the first step of triiodothyronine (T3) and thyroxine (T4) synthesis [5]. Function of iodide transport of NIS has been used in radioiodide imaging and therapy. For well-differentiated thyroid cancer, ^131^I ablation and whole-body scintillation scan after thyroidectomy is currently the most frequent treatment protocol [6]. After NIS gene had been cloned, several investigators used it as an imaging and therapeutic tool in gene therapy. By introducing NIS gene into variety of cancer cells which do not concentrate iodide, it showed a promising therapeutic effect after administrating therapeutic isotopes [7]. By means of *γ*-camera or positron emission tomography with radioiodide, the accumulation of tracer which reflects the expression of transferred NIS gene can be monitored. We have shown the effectiveness of NIS-mediated radioiodide therapy on tumors derived from ATC cell line, ARO [8]. However, the model in previous study was stable cells established by transient transfection and clonal expansion.

Virus-mediated gene delivery has been used for decades in biological research. Retrovirus has been the mainstay of current gene therapy approach due to its high efficiency of infection and high expression of transferred gene. Long-term expression of delivered gene attributed to genome integration is also a useful advantage. However, the use of retrovirus requires the cells being in an actively dividing status [9]. Another type of retrovirus, lentivirus, which infects both dividing and nondividing cells has emerged as potent and versatile vectors for gene transfer [10]. In this study, we aimed to establish a lentiviral system to deliver NIS gene into ARO cells and evaluate the efficacy of radioiodide imaging and therapy.

## 2. Materials and Methods

### 2.1. Cell Culture

293T (BCRC60019) were obtained from the Bioresource Collection and Research Center in Taiwan. The ARO thyroid cancer cell line was kindly provided by Dr. Chin-Wen Chi (National Yang-Ming University, Taiwan). All media and tissue culture supplements were obtained from Gibco-BRL (Invitrogen, Carlsbad, Calif, USA). 293T cells were grown in minimum essential medium (MEM). ARO cells were maintained in RPMI-1640 medium. All complete media were supplemented with 10% fetal bovine serum (FBS), 2 mM L-glutamine, 100 U/mL penicillin, and 100 *μ*g/mL streptomycin. Cells were cultured in a humidified incubator at 37°C and 5% CO_2_: 95% air.

### 2.2. Vector Construction and Virus Production

**Figure 1:**
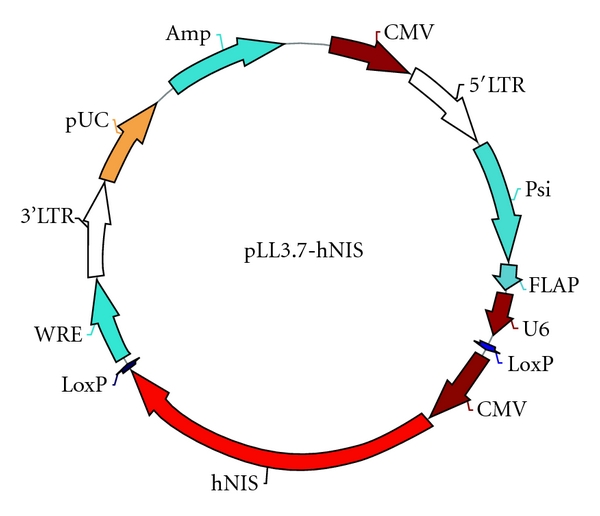
Scheme of constructed hNIS-expressing lentiviral vector.

The hNIS cDNA cloned in pcDNA3 (FL*-hNIS/pcDNA3) was kindly provided by Dr. Sissy Jhiang (Ohio State University). The CMV promoter linked with hNIS in pcDNA3 vector was enzymatically removed and then ligated into LentiLox 3.7 vector (pLL3.7) with proper restriction enzyme to create pLL3.7-hNIS (Figure 1). Lentiviral particles were produced following the protocol published by Tiscornia et al. with some modification [11]. 6 × 10^6^ 293T packaging cells per 100 mm tissue culture plate were seeded and cultured in DMEM supplemented with 10% FBS for 24 hours. Transient transfection was carried out using Lipofectamine 2000 (Invitrogen, Carlsbad, Calif, USA) following the manual instruction. Plasmid DNA and reagent were mixed and incubated for 20 minutes at room temperature to form the DNA-Lipofectamine complexes. The mixture was then added to 293T cell culture and incubated overnight. On the next day, the media were removed and replaced with fresh culture media containing 1% (w/v) BSA. At 72 hour after transfection, the viral particle-containing supernatant was harvested in sterile, capped conical tubes, and centrifuged at 300 g for 15 minutes at 4°C to pellet cell debris. The virus-containing supernatants (LV-CMV-hNIS and LV-CMV-EGFP) were then passed through a sterile, 0.45 *μ*m low protein-binding, Millex-HV PVDF filter (Millipore, Bedford, Mass, USA). The flowthrough was collected and overlaid on the top of 4 mL 20% sucrose solution prepared by PBS in the centrifuging tubes. Virus concentration was carried out by centrifugation at 26.000 rpm, 4°C for 1.5 hrs. After decanting the supernatant, the pellet of viral particles was reconstituted by mixing in the HBSS and stored at −80°C. The virus titer was assayed by the QuickTiter Lentivirus Quantitation Kit (Cell Biolabs, Inc., USA).

### 2.3. Immunocytochemistry Study

Cells were seeded and allowed to grow on 22 mm × 22 mm coverslips (Marienfeld, Lauda-Konigshofen, Germany). After a 24 h incubation, cells were rinsed with PBS for 3 times and then fixed in 4% paraformaldehyde for 15 min at room temperature. After three 5-min washes by PBS, the cells were permeabilized with 0.1% Triton X-100 in PBS/CM/BSA (PBS with 0.1 mM CaCl2, 1 mM MgCl_2_, and 0.2% BSA) for 10 min. After three 5-min washes by PBS, the cells were incubated with a 1 : 100 dilution of mouse anti-hNIS polyclonal antibody (Chemicon, Temecula, USA) for 1 hour at room temperature and washed with PBS. A 1 : 200 dilution of FITC-conjugated goat anti-mouse IgG (Santa Cruz Biotechnology, Santa Cruz, USA) was then added to cells and incubated for 1 hour at room temperature and followed by PBS wash. Nuclei were stained using Bisbenzimide H33342 for 30 minutes at room temperature. After three final PBS washes (5 min each), the cells were mounted with mounting solution. Fluorescent images of cells were observed using a Leica TCS SP5 confocal microscope.

### 2.4. Polymerase Chain Reaction (PCR) for Detecting the Integrated Transgene and Gene Expression

Transduced ARO cells and parental cells were expanded in culture, and total genomic DNA was isolated using the DNeasy kit (Qiagen, Valencia, Calif, USA) following the manufacturer's instruction. Integrated hNIS DNA was amplified by PCR with designed forward primer, 5′-TCAGCACAGCATCCACCAG-3′ and reverse primer, 5′-GGGCACCGTAATAGAGATAG-3′. The PCR protocol was set as follow: 94°C for 4 min, 94°C for 30 s, 56°C for 30 s, and 72°C for 30 s, with repeating reaction cycle for 30 times. For the detection of RNA expression, cells were lysed, and total RNA was isolated using RNeasy mini kit (Qiagen, Valencia, Calif, USA). cDNA synthesis was carried out using MMLV reverse transcriptase (Epicentre Biotechnologies, Madison, Wlis, USA) according to the manufacturer's instruction. In the subsequent PCR reaction, the primers previously described were used to amplify the hNIS gene. As internal controls, primers that hybridize to a segment of the *β*-actin cDNA were included in the same reaction mixture (*β*-actin forward primer: 5′-TCAAGATCATTGCTCCTCCTGAGC-3′; *β*-actin reverse primer: 5′-GGGCACCGTAATAGAGATAG-3′). The annealing temperature for *β*-actin was 54°C.

### 2.5. In Vitro ^125^I Uptake

In vitro cellular ^125^I uptake was measured according to Weiss et al. [12] with little modification. 5 × 10^5^ cells per well were seeded in 12-well plates the day before experiment. After rinsed with Hanks' balanced salt solution (HBSS), cells were incubated with 0.5 mL HBSS containing 10 *μ*M of NaI and 3700 Bq of ^125^I at 37°C. At 5, 10, 20, 30, and 60 minute, cells were washed quickly with 1 mL of iodide-free icecold HBSS. Cells were then detached by trypsin, and all cells were collected and resuspended in 1 mL HBSS in counting tubes. The radioactivity was measured using a Cobra II autogamma counter (Packard instrument, Conn, USA) following the manual procedure.

### 2.6. In Vivo ^124^I Imaging and Quantitative Image Analysis

For in vivo imaging study, 1 × 10^7^ ARO parental cells and cells transduced with LV-CMV-hNIS were subcutaneously injected into the right flank of shoulder of male NOD/SCID mice. After the tumors reached to 8 mm in diameter, the mice were injected intravenously with ^124^I (1.85 MBq). After One hour, mice were anesthetized with 2% isoflurane (Forane Abbott Laboratories, Queenboroug, Kent, England) and 98% oxygen mixture and secured on the imaging table in the prone position. Static images were obtained using the microPET R4 scanner (Siemens Preclinical Solutions, Knoxville, Tenn, USA) for 30 min. Fully 3-dimensional list mode data were collected for 30 min using an energy window of 350–750 keV and a timing window of 6 ns. For maximum sensitivity, coincidence data were rebinned into 3-dimensional sinograms using the full axial acceptance angle of the scanner (max. ring difference = 31). To preserve axial resolution, high sampling of the polar angle was used (span = 3). The resulting 3D sinograms were then rebinned into 2D sinograms using Fourier rebinning (FORE) and reconstructed with 2-dimensional filtered back projection (FBP) using a ramp filter with cutoff at Nyquist. The image matrix size was 256 × 256. Data manipulation was carried out via the MicroPET Manager software provided by the manufacturer. No attenuation correction was performed. The images were interpreted qualitatively. All images were expressed in the ordinary rainbow color scale with standard visual spectrum installed in the computer of the microPET system.

### 2.7. In Vivo ^131^I Therapy

Tumors grown on mice were assessed for the effect of ^131^I therapy. Two weeks after cell inoculation, mice bearing tumors were intraperitoneally injected with 55.5 MBq (1.5 mCi) ^131^I or same volume of normal. The tumor size was measured twice a week after ^131^I treatment. Tumor volume was measured by caliper and calculated using the equation *VT*  (mm^3^) = *L* × *W* × *D* × 0.52, where *VT* is the estimated tumor volume, and *L*, *W*, and *D* are the length (mm), width (mm), and depth (mm) of the tumor, respectively.

## 3. Result

### 3.1. Immunocytochemistry Study

**Figure 2:**
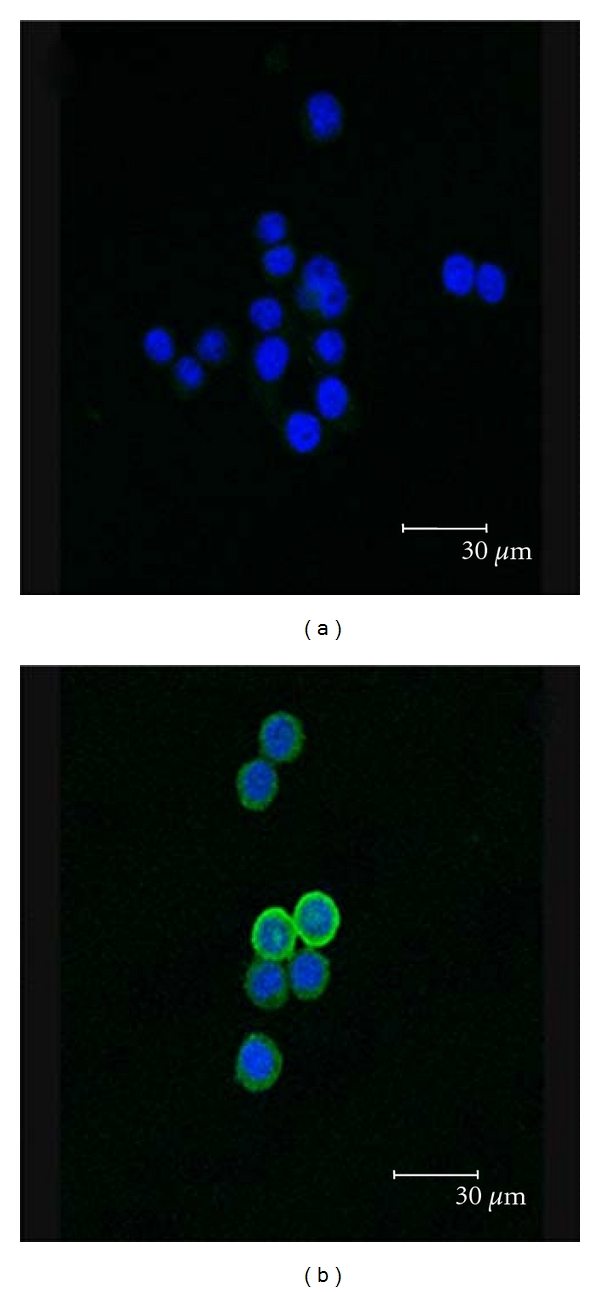
Immunofluorescence of ARO cells transduced with LV-CMV-hNIS. Cells were immunostained with mouse anti-hNIS antibody and FITC-conjugated goat anti-mouse 2nd antibody. ARO parental cells showed no detection of hNIS expression (a). Nuclei was stained by H33342 and shown in blue. hNIS protein expression (green) on the cellular membrane of transduced ARO cells was clearly detected by confocal fluorescent microscopy (b).

To evaluate the hNIS expression in transduced ARO cells, we performed immunofluorescent staining. Using mouse anti-hNIS primary antibody coupled with FITC-conjugated goat anti mouse IgG, fluorescence emitted from FITC fluorophore was shown localized on the cellular surface of transduced cells, compared with the location of cell nuclei stained by H33342 (Figure 2(b)). ARO parental cells treated with the same staining procedure showed no FITC-related fluorescent signal, indicating no or low expression of hNIS in these cells (Figure 2(a)). This result indicates the efficient transfer and subsequent expression of hNIS by lentiviral transduction.

### 3.2. PCR for Detecting the Integrated Viral Vector and Gene Expression

**Figure 3:**
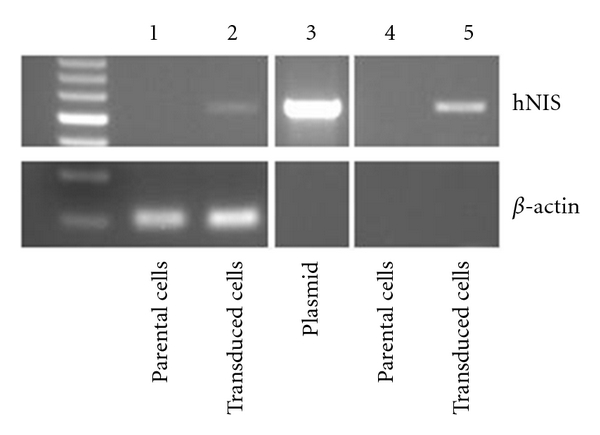
Detection of integrated hNIS sequence and RNA expression Lentiviral integration of hNIS construct was confirmed by PCR amplifying the specific DNA fragment in genomic DNA. Lane 4 and 5 represent the result of amplifying integrated hNIS fragment from isolated genomic DNA of parental and transduced cells, respectively. As assayed by RT-PCR, lane 1 and 2 represent the RNA expression of exogenous hNIS of parental and transduced cells, respectively. Same reaction was also carried out for detecting RNA expression of *β*-actin and was shown in all lanes. Lane 3 represents the PCR result with hNIS-containing plasmid as the template which was a control.

To verify the lentiviral integration of exogenous hNIS construct in transduced ARO cells, we have previously performed PCR with designed primers to detect the integrated construct in genomic DNA isolated from ARO parental cells and transduced cells [13]. In Figure 3, transduced cells showed apparent exogenous hNIS PCR amplicon with expected size (lane 5) while parental cells had no detected band (lane 4). RT-PCR was also performed to detect RNA expression in parental and transduced cells. After performing reverse transcription and subsequent PCR, expression of exogenous hNIS was observed (lane 2) and parental cells showed no detected expression (lane 1).

### 3.3. In Vitro Radioiodide Uptake

**Figure 4:**
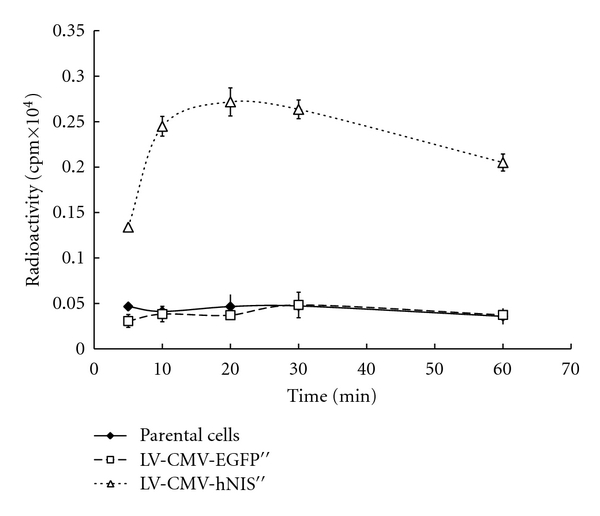
Time course of ^125^I uptake of ARO cells. ARO parental cells, cells transduced with LV-CMV-EGFP or LV-CMV-hNIS were measured for accumulation of ^125^I at different time point. ARO cells transduced with LV-CMV-hNIS showed more effective accumulation of ^125^I than parental cells or cells transduced with LV-EGFP. Values are the mean ± standard deviation (*n* = 3).

To evaluate the function of iodide uptake of transduced cells, we have previously performed the sequential in vitro ^125^I uptake experiment [13]. ARO parental cell or cells transduced with LV-CMV-hNIS or LV-CMV-hNIS were incubated in ^125^I-containing HBSS followed by *γ* counting the radioactivity within cells at different time points. As shown in Figure 4, cells transduced with LV-CMV-hNIS accumulated ^125^I rapidly in 10 minutes, and the radioactivity reached to the plateau at 20 minutes from beginning. ARO parental cells and cells transduced with LV-CMV-EGFP showed a minimal ^125^I uptake and no higher accumulation as the time increased. At 20 minute, the index of accumulated ^125^I in cells transduced with LV-CMV-hNIS was ~7 times higher than that of parental cells or cells transduced with LV-CMV-EGFP. This result demonstrates the lentiviral-transferred hNIS, exhibits its original function, and consequently restores the ability of iodide uptake of anaplastic thyroid cancer cells.

### 3.4. In Vivo Radioiodide Imaging by MicroPET

**Figure 5:**
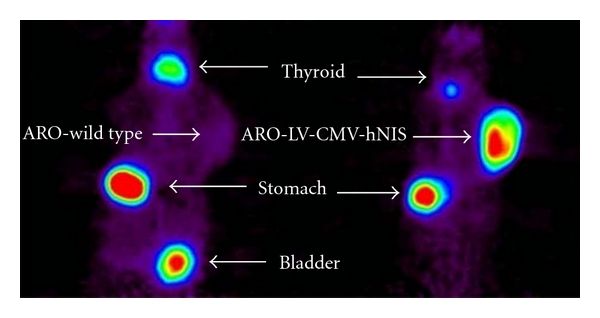
In vivo ^124^I MicroPET imaging ARO parental cells and cells transduced with LV-CMV-hNIS were subcutaneously implanted into mice shoulder and allowed to form the tumors. When tumors reached 8 mm in size, images were taken for 30 min at 60 min after administration of 1.85 MBq (50uCi) of ^124^I. Images show that tumor derived from transduced ARO cells accumulates ^124^I more efficiently than tumor derived from ARO cells.

In order to know whether ARO-transduced cells retain the expression of hNIS and the function of iodide uptake, we established the tumors derived from ARO parental and transduced cells on NOD/SCID mice. 1 hour after intravenously injecting ^124^I, mice were imaged using microPET scanner. Static image of mice bearing tumor derived from ARO parental cells (left) and transduced cells (right) was acquired for 30 minutes and is shown in Figure 5. Tumor derived from transduced cells showed more efficient accumulation of ^124^I while compared to tumor derived from parental cells. This result indicates transduced cells retain not only the expression of transferred hNIS but the ability to accumulate iodide.

### 3.5. In Vivo ^131^I Therapy

**Figure 6:**
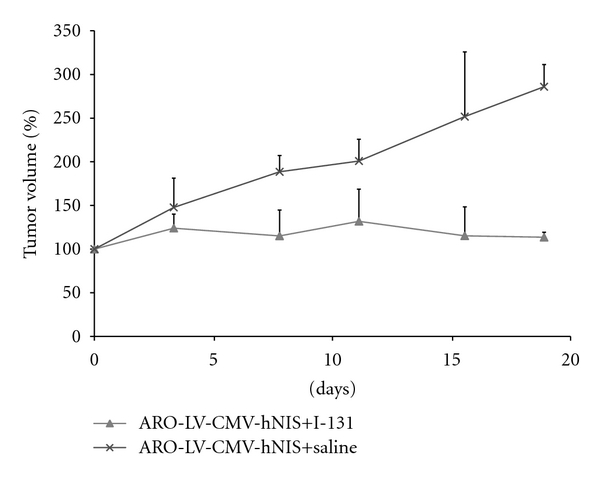
In vivo ^131^I therapy of ARO tumors mice bearing tumors derived from transduced ARO cells were intraperitoneally injected with 55.5 MBq (1.5 mCi) of ^131^I or same volume of saline. Tumor growth was recorded twice a week. The group treated with ^131^I (*n* = 5) showed efficient inhibition of tumor growth while the group treated with saline (*n* = 3) showed rapid growth of tumors.

To know the efficacy of ^131^I therapy of hNIS-expressing tumor, mice bearing tumors were treated with 55.5 MBq (1.5 mCi) of ^131^I or saline. Result shows that tumor growth was effectively inhibited by treating with ^131^I, as compared to tumors treated with saline (Figure 6). This data as well as result of in vivo imaging indicate tumor derived from transduced cells that retain the expression and function of hNIS even in the in vivo environment.

## 4. Discussion

Using lentiviral vector, we have successfully transduced ATC cells with NIS gene which was markedly expressed and well functioning in the transduced cells. Tumors established by transduced cells retained high expression of hNIS which enable tumors to be well treated by ^131^I and monitored with radioiodide imaging. Our previous data showed that stable cells offered a good model to test the feasibility of NIS-mediated gene therapy. However, gene delivery by viral transduction is more applicable in vivo. Lentivitral gene transfer was intensively used due to its advantages, including stable integration of transgene, capability of transducing dividing and nondividing cells and long-term transgene expression. Kim et al. showed effective long-term monitoring and radionuclide therapy of colon cancer cells in living organism by using a lentiviral vector system carrying hNIS controlled by UbC promoter [14]. Dingli et al. also used lentivectors to target and express hNIS in myeloma cells for ^131^I therapy. Results showed that tumor xenografts treated with ^131^I were completely eradicated without recurrence [15]. Reports above indicate the potential of lentiviral vector combined with NIS gene therapy on cancer. Our data is the first demonstration of lentiviral NIS-mediated gene therapy on anaplastic thyroid cancer.

Iodide is retained within thyroid gland for a period of time due to multiple processes of thyroid hormone synthesis. Zuckier et al. reported that ^131^I concentrated within thyroid gland continued to increase at 19 h after administration into mice according to a biodistribution experiment [16]. Same result was noted by our previous study [8]. By analyzing ROIs of serial images acquired using a *γ*-camera, radioactivity in thyroid gland reached the peak around 20 hours after ^131^I injection. Similar experiment also revealed that within 5 hours from injection, equal level of ^131^I accumulation was noted in thyroid gland and in tumors derived from ARO stable cells, while only 1/3 of ^131^I activity was measured in tumors derived from ARO parental cells. Radioactivity within tumors decreased after 5 hours in contrast to increase in thyroid gland. Results showed that without further steps of T3 and T4 synthesis, iodide pumped into cell by NIS is rapidly effluxed. This drawback greatly reduces the feasibility of NIS in gene therapy.

To solve the problem of short retention of radioiodide, Huang et al. coexpressed NIS and TPO in nonsmall cell lung cancer which increased retention time of radioiodide within cells as compared to cells expressing NIS alone [17]. Furuya et al. delivered TTF-1 (thyroid transcription factor 1) to NIS-transfected cells. TTF-1 is the thyroid specific transcription factor and able to turn on the expression of downstream thyroid specific genes such as Tg, TPO, and NIS [18–20]. However, delivery of more than one gene into cells greatly increases the difficulty. Despite the short retention of radioiodine within transduced cells, the growth of tumors still showed significant inhibition in this study and our previous data [8]. Highly expressed NIS alone with sufficient but nonlethal dose of administered ^131^I may compensate the problem of short retention of radioiodide.

Recently, a comprehensive DNA sequence profiling analysis has pointed that ARO cells are highly identical with HT-29, a colon cancer cell line [21]. However, several studies demonstrated that with treatment of certain agents, expression of thyroid specific genes could be restored in ARO cells. Landriscina et al. showed that the reverse transcriptase inhibitors recovered the thyrotropin signaling and iodide uptake ability [22]. Upon treatment of histone deacetylase (HDAC) inhibitors such as depsipeptide and trichostatin A (TSA), thyroid-specific gene expression and accumulation of radioiodide through the iodination of generic cellular proteins were detected in ARO cells [23]. ARO cells were also shown to express thyroid stimulatory hormone receptor (TSHR), and the population which expressed TSHR increased following treatment of TSH in a dose-dependent manner [24]. Despite the published results which demonstrated that ARO cells retain the function and specific gene expression related to thyroid cells, more evidences are needed to clarify the identity of ARO cells.

Lentiviral gene transfer is considered as a promising approach to cancer gene therapy. Cancer-specific targeting is one of key point in the in vivo gene therapy. In this study, CMV promoter which has been reported as one of the strongest promoters in vitro was used in our transduction system. However, in the in vivo system, silence of gene governed by CMV was noted within a few weeks in several organs [25, 26]. In addition, CMV is a universal promoter which cannot be used to specifically activate downstream gene expression in specific cell type. Therefore, a promoter which specifically target ATC is urgently needed. We previously demonstrated that using a hepatoma chimeric promoter (EIIA enhancer combined with *α*-fetoprotein promoter) to target hepatocarcinoma cells (HCCs) resulted in specifically turning on the hNIS transgene in HCC, but not in non-HCC [13]. Thyroglobulin promoter was reported to target thyroid cancer. With transfer and specific expression of TK gene driven by the Tg promoter in thyroid cancer cells, result showed marked killing ability in the presence of GCV, little in vivo toxicity, and should be useful in the future for treating thyroid Tg-producing cancers [27]. ATC is known as a poorly differentiated thyroid carcinoma with reduced or silenced expression of several thyroid-specific genes, including NIS and Tg. Therefore, to design an ATC-specific lentiviral targeting system, promoter of Tg gene can not work for this purpose. Our future work will focus on searching for a suitable promoter for ATC in our lentivirus-mediated NIS radiogene therapy.

## 5. Conclusion

We have established a lentiviral system for delivering hNIS to ARO cell. The transduced cells showed high expression of the transgene and restored the ability of radioiodide accumulation. Tumors derived from the transduced cells showed effective in vivo imaging and therapy. This study offers a promising, applicable way to treat ATC.

## References

[1] Pasieka JL (2003). Anaplastic thyroid cancer. *Current Opinion in Oncology*.

[2] Are C, Shaha AR (2006). Anaplastic thyroid carcinoma: biology, pathogenesis, prognostic factors, and treatment approaches. *Annals of Surgical Oncology*.

[3] Giuffrida D, Gharib H (2000). Anaplastic thyroid carcinoma: current diagnosis and treatment. *Annals of Oncology*.

[4] Dohan O, De La Vieja A, Paroder V (2003). The sodium/iodide symporter (NIS): characterization, regulation, and medical significance. *Endocrine Reviews*.

[5] Dohan O, Portulano C, Basquin C, Reyna-Neyra A, Amzel LM, Carrasco N (2007). The Na+/I- symporter (NIS) mediates electroneutral active transport of the environmental pollutant perchlorate. *Proceedings of the National Academy of Sciences of the United States of America*.

[6] Woodrum DT, Gauger PG (2005). Role of 131I in the treatment of well differentiated thyroid cancer. *Journal of Surgical Oncology*.

[7] Hingorani M, Spitzweg C, Vassaux G (2010). The biology of the sodium iodide symporter and its potential for targeted gene delivery. *Current Cancer Drug Targets*.

[8] Hsieh YJ, Ke CC, Liu RS (2007). Radioiodide imaging and treatment of ARO cancer xenograft in a mouse model after expression of human sodium iodide symporter. *Anticancer Research*.

[9] Maier P, Von Kalle C, Laufs S (2010). Retroviral vectors for gene therapy. *Future Microbiology*.

[10] Matrai J, Chuah MK, Vandendriessche T (2010). Recent advances in lentiviral vector development and applications. *Molecular Therapy*.

[11] Tiscornia G, Singer O, Verma IM (2006). Production and purification of lentiviral vectors. *Nature Protocols*.

[12] Weiss SJ, Philp NJ, Grollman EF (1984). Iodide transport in a continuous line of cultured cells from rat thyroid. *Endocrinology*.

[13] Liu RS, Hsieh YJ, Ke CC (2009). Specific activation of sodium iodide symporter gene in hepatoma using alpha-fetoprotein promoter combined with hepatitis B virus enhancer (EIIAPA). *Anticancer Research*.

[14] Hyun JK, Yong HJ, Joo HK (2007). In vivo long-term imaging and radioiodine therapy by sodium-iodide symporter gene expression using a lentiviral system containing ubiquitin C promoter. *Cancer Biology and Therapy*.

[15] Dingli D, Diaz RM, Bergert ER, O’Connor MK, Morris JC, Russell SJ (2003). Genetically targeted radiotherapy for multiple myeloma. *Blood*.

[16] Zuckier LS, Dohan O, Li Y, Chee JC, Carrasco N, Dadachova E (2004). Kinetics of perrhenate uptake and comparative biodistribution of perrhenate, pertechnetate, and iodide by NaI symporter-expressing tissues in vivo. *Journal of Nuclear Medicine*.

[17] Huang M, Batra RK, Kogai T (2001). Ectopic expression of the thyroperoxidase gene augments radioiodide uptake and retention mediated by the sodium iodide symporter in non-small cell lung cancer. *Cancer Gene Therapy*.

[18] Damante G, Di Lauro R (1994). Thyroid-specific gene expression. *Biochimica et Biophysica Acta*.

[19] Guazzi S, Price M, De Felice M, Damante G, Mattei MG, Di Lauro R (1990). Thyroid nuclear factor 1 (TTF-1) contains a homeodomain and displays a novel DNA binding specificity. *EMBO Journal*.

[20] Kimura S (1997). Role of the thyroid-specific enhancer-binding protein in transcription, development and differentiation. *European Journal of Endocrinology*.

[21] Schweppe RE, Klopper JP, Korch C (2008). Deoxyribonucleic acid profiling analysis of 40 human thyroid cancer cell lines reveals cross-contamination resulting in cell line redundancy and misidentification. *Journal of Clinical Endocrinology and Metabolism*.

[22] Landriscina M, Fabiano A, Altamura S (2005). Reverse transcriptase inhibitors down-regulate cell proliferation in vitro and in vivo and restore thyrotropin signaling and iodine uptake in human thyroid anaplastic carcinoma. *Journal of Clinical Endocrinology and Metabolism*.

[23] Furuya F, Shimura H, Suzuki H (2004). Histone deacetylase inhibitors restore radioiodide uptake and retention in poorly differentiated and anaplastic thyroid cancer cells by expression of the sodium/iodide symporter thyroperoxidase and thyroglobulin. *Endocrinology*.

[24] Friedman S, Lu M, Schultz A, Thomas D, Lin RY (2009). CD133+ anaplastic thyroid cancer cells initiate tumors in immunodeficient mice and are regulated by thyrotropin. *PLoS ONE*.

[25] Baskar JF, Smith PP, Nilaver G (1996). The enhancer domain of the human cytomegalovirus major immediate-early promoter determines cell type-specific expression in transgenic mice. *Journal of Virology*.

[26] Loser P (1998). Reactivation of the previously silenced cytomegalovirus major immediate-early promoter in the mouse liver: involvement of NFkappaB. *Journal of Virology*.

[27] Zhang R, Straus FH, DeGroot LJ (2001). Adenoviral-mediated gene therapy for thyroid carcinoma using thymidine kinase controlled by thyroglobulin promoter demonstrates high specificity and low toxicity. *Thyroid*.

